# Functions and clinical significance of circular RNAs in acute myeloid leukemia

**DOI:** 10.3389/fphar.2022.1010579

**Published:** 2022-11-24

**Authors:** Min Zhou, Xianling Gao, Xin Zheng, Jing Luo

**Affiliations:** ^1^ School of Life Sciences, Chongqing University, Chongqing, China; ^2^ Center of Plant Functional Genomics, Institute of Advanced Interdisciplinary Studies, Chongqing University, Chongqing, China; ^3^ Department of Anesthesiology, First Affiliated Hospital of Kunming Medical University, Kunming, China; ^4^ Department of Pharmacy, West China Hospital, Sichuan University, Chengdu, China; ^5^ Department of Anesthesiology, The First People’s Hospital of Yunnan Province, Kunming, China; ^6^ Department of Anesthesiology, The Affiliated Hospital of Kunming University of Science and Technology, Kunming, China

**Keywords:** circular RNAs, acute myeloid leukemia, molecular functions, clinical significance, role

## Abstract

Circular RNAs (circRNAs) are a class of covalently closed single-stranded RNA molecules. Four types of circRNAs have been reported in animal cells, and they have typical characteristics in their biogenesis, nuclear export and degradation. Advances in our understanding of the molecular functions of circRNAs in sponging microRNAs, modulating transcription, regulating RNA-binding proteins, as well as encoding proteins have been made very recently. Dysregulated circRNAs are associated with human diseases such as acute myeloid leukemia (AML). In this review, we focus on the recently described mechanisms, role and clinical significance of circRNAs in AML. Although great progress of circRNAs in AML has been achieved, substantial efforts are still required to explore whether circRNAs exert their biological function by other mechanisms such as regulation of gene transcription or serving as translation template in AML. It is also urgent that researchers study the machineries regulating circRNAs fate, the downstream effectors of circRNAs modulatory networks, and the clinical application of circRNAs in AML.

## Introduction

Acute myeloid leukemia (AML) is a hematological malignancy characterized by clonal expansion of myeloid blasts cells with uncontrolled proliferation in the bone marrow and peripheral blood (Almatani et al., 2021; Aung et al., 2021; Andreozzi et al., 2022). AML has become a central research focus because it is the most common type of acute leukemia in adults worldwide, with rising morbidity and mortality (Gallipoli et al., 2015; Liu et al., 2019; Bhattacharya and Gutti 2022). The key therapeutic strategies for AML include chemotherapy, allogenic hematopoietic stem cell transplantation and palliative care (Xiang et al., 2022). However, although these advancements in the treatment of AML, the overall prognosis is poor (5-yeal overall survival only 28.7%) (Singh V. et al., 2021). Thus, new biomarker and precision therapy method are urgent to be found for the treatment of AML.

**FIGURE 1:**
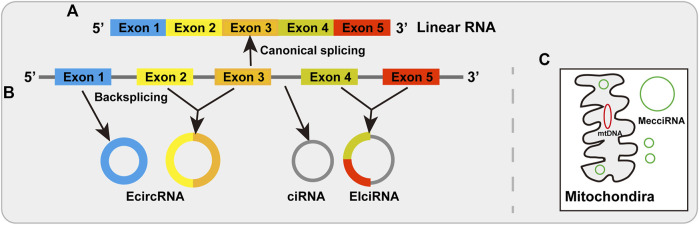
Biogenesis of linear RNAs and circRNAs. **(A)** Linear RNAs are produced by canonical splicing *via* joining an upstream 5′ splicing site to a downstream 3′ splicing site. **(B)** The biogenesis of circRNAs. EcircRNAs are produced by backsplicing and distribute predominantly in the cytoplasm. EIciRNAs are generated by intronic sequences retained between the backsplicing exons that are distributed mainly in the nucleus. CiRNAs from intronic lariat RNA precursors that distribute in the nucleus. **(C)** MecciRNAs are mitochondria-encoded circRNAs that are found in the cytosol and mitochondria.

By the splicing machinery in linear order, most eukaryotic genes were divided by introns which must be removed from precursor message RNA *via* linking an upstream 5′ splicing site to a downstream 3′ splicing site (Wahl et al., 2009; Wilusz 2018) (Figure 1A). However, it has been reported that the splicing event can also occur in a non-canonical way to make the backsplicing reaction by linking a downstream 5′ splicing site to an upstream 3′ splicing site, thereby producing circular RNAs (Nigro et al., 1991; Cocquerelle et al., 1993; Chen et al., 2015; Wilusz 2017; Chen 2020). Circular RNAs (circRNAs), typically produced from protein-encoding genes through backsplicing, are a large class of covalently closed single-stranded RNA molecules, without a 5′ end or a 3’ poly (A) tail (Wilusz 2018; Kristensen et al., 2019; Papatsirou et al., 2021; Zhou et al., 2021) (Figure 1B). There are four types circRNAs in animal cells, including exonic circRNAs (EcircRNAs), exon-intron circRNAs (EIciRNAs), and intronic circRNAs (ciRNAs) (Jeck et al., 2013; Chen et al., 2015; Wilusz 2018; Chen 2020; Chen L. et al., 2022) (Figure 1B). Recently another type has been reported as mitochondria-encoded circRNAs (mecciRNA) (Liu et al., 2020) (Figure 1C). Although some circRNAs (such as EIciRNAs and ciRNAs) have been found in the nucleus (Li Z. et al., 2015; Conn et al., 2017), most circRNAs (EcircRNAs) are export to the cytoplasm (Salzman et al., 2012; Jeck et al., 2013). With the development of high-throughput RNA sequencing, many circRNAs have been identified from protein-coding genes across different species, tissues and cell lines (Memczak et al., 2013; Chen et al., 2015; Wang et al., 2016; Wilusz 2018; Zheng et al., 2021). CircRNAs have been found to play vital roles in different molecular and cellular events through different mechanisms including acting as microRNA sponges, regulation of transcription, interacting with RNA binding proteins and serving as translation template (Kristensen et al., 2019; Li et al., 2020b; Ma et al., 2020; Singh D. et al., 2021; Zheng et al., 2021; Zhou et al., 2021).

With the development of study methods, our understanding of the general characteristics of circRNAs and their functions in normal physiology and most human diseases has been improved. Here, we focus on the recently described functional relevance of individual circRNAs to leukemia and their clinical significance. We first offer a brief introduction to the mechanisms of circRNAs, and then focus on recently described their roles and clinical significance in AML.

## Mechanisms of circRNAs

**FIGURE 2:**
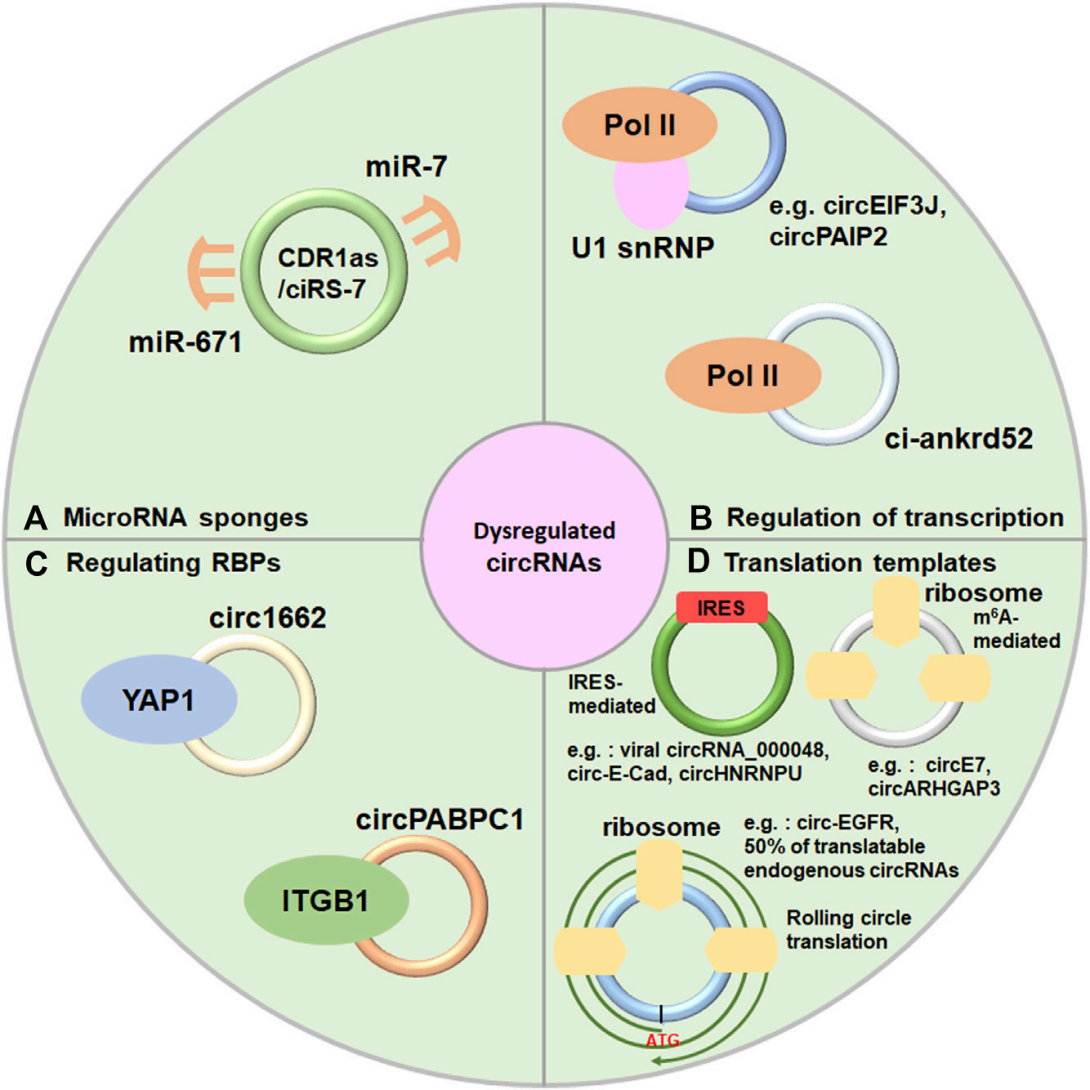
Diverse mechanisms of circRNAs. **(A)** Acting as microRNAs sponges, **(B)** Regulation of transcription. **(C)** Interacting with RNA binding proteins. **(D)** Serving as translation templates.

Although the functions of most circRNAs are not fully explored, emerging evidence is beginning to uncover that dysregulated circRNAs play invital roles in many biological processes as regulatory noncoding RNAs, such as acting as microRNAs, regulating transcription, interacting with RNA binding proteins (Figures 2A–C). A part of circRNAs are also recognized as regulatory coding RNAs encoding small functional peptides (Figure 2D).

## Acting as microRNA sponges

Numerous researches have indicated that circRNAs have one or more microRNA binding sites and serve as microRNA sponges to prevent microRNAs away from their target genes which have been well-documented by researchers (D'Ambra et al., 2019; Kristensen et al., 2019; Zhou et al., 2021). For instance, CDR1as/ciRS-7 is one of the classic and most researched circRNAs which contains one miR-671 binding site and over 60 conserved miR-7 binding sites (Memczak et al., 2013) (Figure 2A). In addition, circBIRC6 contains miR-145 and miR-34a binding sites to regulate human cell pluripotency (Yu et al., 2017). Some other circRNAs also can sponge microRNAs, despite the majority of them only have a limited amount of microRNA binding sites (Panda 2018; Qiu et al., 2021; Shen et al., 2022). Further researches are required to identify in what extend circRNAs could sponge microRNAs due to the lower expression of circRNAs.

## Regulation of transcription

Nucleus-localized circRNAs (EIciRNAs and ciRNAs) and some EcircRNAs were proposed to play important role in transcriptional regulation. EIcircRNAs such as circEIF3J and circPAIP2, facilitate transcription initiation by RNA polymerasemer II (Pol II) at the promoter of host gene through recruiting U1 small nuclear ribonucleoprotein (U1 snRNP) in human cells (Li Z. et al., 2015) (Figure 2B). Furthermore, ci-ankrd52, a ciRNA, produces an R-loop in cis and facilitates transcription elongation *via* Pol II (Zhang et al., 2013; Li X. et al., 2021) (Figure 2B). Furthermore, some EcircRNAs also modulate transcription through interacting with chromatin. For instance, circFECR1 promotes FLI1 transcription in cis through recruting the TET1 (a demethylase) to result in DNA demethylation (Chen N. F. et al., 2018), and circSMARCA5 forms an R-loop by binding to its parent gene locus, resulting in transcriptional pausing at exon 15 of SMARCA5 (Xu et al., 2020).

## Interacting with RNA binding proteins

Numerous studies indicated that circRNAs were also found to reveal different functions through directly interacting with different proteins (Holdt et al., 2016; Abdelmohsen et al., 2017; Li X. et al., 2017; Wu et al., 2019; Shen et al., 2020; Chen C. et al., 2021; Shi et al., 2021). For instance, Circ1662 accelerated the nuclear transport of TAP1 by restraining YAP1 phosphorylation (Chen C. et al., 2021) (Figure 2C). In a ubiquitination-dependent manner, circPABPC1 directly linked ITGB1 to the 26S proteasome for degradation in liver cancer (Shi et al., 2021) (Figure 2C). Furthermore, circECE1 prevented speckle-type POZ-mediated c-Myc ubiquitination and degradation through interacting with c-Myc in osteosarcoma (Shen et al., 2020). In addition, circYAP negatively modulated YAP expression by inhibiting the assembly of the YAP translation inititaion marinery in breast cancer cells (Wu et al., 2019).

## Serving as translation template

CircRNAs have been regarded as a class of non-coding RNAs for long periods of time. However, recent researches have revealed that circRNA can be translated into functional peptides (Legnini et al., 2017; Liu Y. et al., 2021; Wu et al., 2021; Liu et al., 2022; Zhang et al., 2022) (Table 1). CircRNAs could be translated into proteins through internal ribosome entry site (IRES) in a cap-independent manner, m^6^A-dependent initiation of translation and rolling circle translation (Figure 2D). For instance, endogenous circRNAs, such as viral circRNA_000048, circ-E-Cad and circHNRNPU, can translate to a micropeptide vsp21, C-E-Cad-254aa and MAPK1-109aa through IRES-dependent manner (Gao et al., 2021; Tang et al., 2022; Zhang et al., 2022) (Figure 2D), respectively. Moreover, circE7 and circARHGAP3 can translate to E7 protein and circARHGAP35-encoded protein by m^6^A-dependent initiation of translation (Zhao et al., 2019; Li Y. et al., 2021) (Figure 2D), respectively. Furthermore, circ-EGFR can translate to rolling-translated-EGFR through rolling circle translation (Liu Y. et al., 2021) (Figure 2D), respectively. In addition, Fan et al. recently reported that 50% of translatable endogenous circRNAs experience rolling circle translation, several of which are experimentally verfied (Fan et al., 2022) (Figure 2D). For instance, mutation of the IRES-like element (AAGAAG) in circPFAS decrease its translation (Fan et al., 2022).

**TABLE 1:** Reported translatable circRNAs.

CircRNAs	Proteins	Found in	Mechanism	References
Viral curcRNA_000048	Micropeptide vsp21	*Bombyx mori cypovirus*	IRES in a cap-independent manner	Zhang et al. (2022)
Circ-E-Cad	C-E-Cad-254aa	Glioblastoma	IRES in a cap-independent manner	Gao et al. (2021)
CircHNRNPU	CircHNRNPU-603aa	Mutiple myeloma	IRES in a cap-independent manner	Tang et al. (2022)
CircMAPK1	MAPK1-109aa	Gastric cancer	IRES in a cap-independent manner	Jiang et al. (2021)
Circ-SMO	SMO-193aa	Glioblastoma	IRES in a cap-independent manner	Wu et al. (2021)
CircDIDO1	DIDO1-529aa	Gastric cancer	IRES in a cap-independent manner	Zhang Y. et al. (2021)
Hsa_circ_0006401	Hsa_circ_0006401 peptide-198aa	Colorectal cancer	IRES in a cap-independent manner	Zhang C. J. et al. (2021)
CircHER2	HER2-103aa	Triple negative breast cancer	IRES in a cap-independent manner	Li et al. (2020a)
CircAβ-a	Aβ175	Alzheimer’s disease	IRES in a cap-independent manner	Mo et al. (2020)
Circ-AKT3	AKT3-174aa	Gliobastoma	IRES in a cap-independent manner	Xia et al. (2019)
CircPPP1R12A	PPP1R12A-73aa	Colon cancer	IRES in a cap-independent manner	Zheng et al. (2019)
Circ-SHPRH	SHPRH-146aa	Gliobastoma	IRES in a cap-independent manner	Zhang et al. (2018a)
CircPINTexon2	PINT-87aa	Gliobastoma	IRES in a cap-independent manner	Zhang et al. (2018b)
Circ-ZNF609	ZNF609-250aa	Myogenesis	IRES in a cap-independent manner	Legnini et al. (2017)
Circβ-catenin	β-catenin isoform-370 aa	Hepatocellular carcinoma	IRES in a cap-independent manner	Liang et al. (2019)
CircMbl1	Circ-Mbl1-encoded protein	Fly head	IRES in a cap-independent manner	Pamudurti et al. (2017)
Circ-FBXW7	FBXW7-185aa	Gliobastoma	IRES in a cap-independent manner	Yang et al. (2018)
CircMbl3	Circ-Mbl3-encoded protein	Fly head	IRES in a cap-independent manner	Pamudurti et al. (2017)
CircE7	E7 protein	High-risk HPV	m^6^A-dependent initiation of translation	Zhao et al. (2019)
CircARHGAP35	CircARHGAP35-encoded protein	Hepatocellular carcinoma	m^6^A-dependent initiation of translation	Li Y. et al. (2021)
CircMET	MET404	Glioblastoma	m^6^A-dependent initiation of translation	Zhong et al. (2022)
Circ-EGFR	Rolling-translated-EGFR	Glioblastoma	Rolling circle translation	Liu Y. et al. (2021)
50% of translatable endogenous circRNAs	NA	293T	Rolling circle translation	Fan et al. (2022)
CircSfl	CircSfl-encoded peptide	Fly brain and muscle	NA	Weigelt et al. (2020)
CircGprc5a	CircGprc5a-peptide	Bladder cancer stem cells	NA	Gu et al. (2018)

## The role of circRNAs in acute myeloid leukemia

The role of circRNAs in AML biology and pathogenesis has been investigated (Table 2). Increasing evidenence shows that circRNAs play important role in gene expression and regulate distinct steps of leukemogenesis, such as differentiation, cell cycle progress, proliferation and apoptosis (Jamal et al., 2019; Singh V. et al., 2021). They also involve in drug resistance in AML chemotherapy (Shang et al., 2019; Li M. et al., 2020; Ding et al., 2021). The role of circRNAs in AML will be discussed in the following sections.

**TABLE 2:** Dysregulated circRNAs and their function in AML.

CircRNA	Expression	Molecular mechaism	Biological function	References
Circ_00059707	Down	Regulaing miR-1287-5p	Regulation of cell growth and apoptosis	Ma et al. (2022)
Cir_POLA2	Up	Regulating miR-34a	Regulation of cell proliferation	Li H. et al. (2021)
CircSPI1	Up	Sponging miR-1307-3p/miR-382-5p/miR-767-5p	Oncogene, regulation of cell proliferation and apoptosis	Wang X. L. et al. (2021)
Hsa_circ_0012152	Up	Regulating miR-625-5p/SOX12 axis	Regulation of cell proliferation and apoptosis	Shang et al. (2021)
Hsa_circ_0002483	Up	Regulating miR-758-3p/MYC axis	Regulation of cell proliferation and cell cycle arrest and apoptosis	Xiao et al. (2021)
Circ_PTK2	Up	Regulating miR-330-5p/FOXM1 axis	Regulation of cell proliferation and apoptosis	Yi et al. (2021)
Hsa_circ_0121,582	Down	Regulation of miR-224/GSK3β	Inhibition of cell proliferation	Chen et al. (2020)
Hsa_circ_0000370	Up	Regulating of miR-1299 and S100 calcium-binding protein A7A protein	Regulation of cell viability and apoptosis	Zhang L. et al. (2020)
CircRNA RNF13	Up	Regulating miR-1224-5p	Regulation of cell proliferation, migration and apoptosis, and cell cycle progression	Zhang R. et al. (2020)
Hsa_circ_0009910	Up	Regulating miR-20-5p	Regulation of cell proliferation and apoptosis	Ping et al. (2019)
Hsa_circRNA-100290	Up	Regulation of miR-203/Rab10 axis	Regulation of cell proliferation and apoptosis	Fan et al. (2018)
Circ-DLEU2	Up	Regulating of miR496/PRKACB axis	Regulation of cell proliferationn and apoptosis	Wu et al. (2018)
CircSPI1	Up	Regulating Eif4AIII and PU.1	Regulation of myeloid differentiation of AML cells	Wang X. L. et al. (2021)
CirPLXNB2	Up	Regulation of PLXNB2, BCL2, cyclin D1 and BAX	Regulation of cell proliferation, migration and apoptosis	Lin et al. (2021)
CircMYBL2	Up	Upregulation of FLT3 translation	Regulation of cell proliferation and apoptosis, and cell cycle progression	Sun et al. (2019)
CircBCL11B	Up	NA	Regulation of leukemic cell proliferation	Lux et al. (2021)

## Dysregulation of circRNAs in acute myeloid leukemia and their association with acute myeloid leukemia phenotype

**FIGURE 3:**
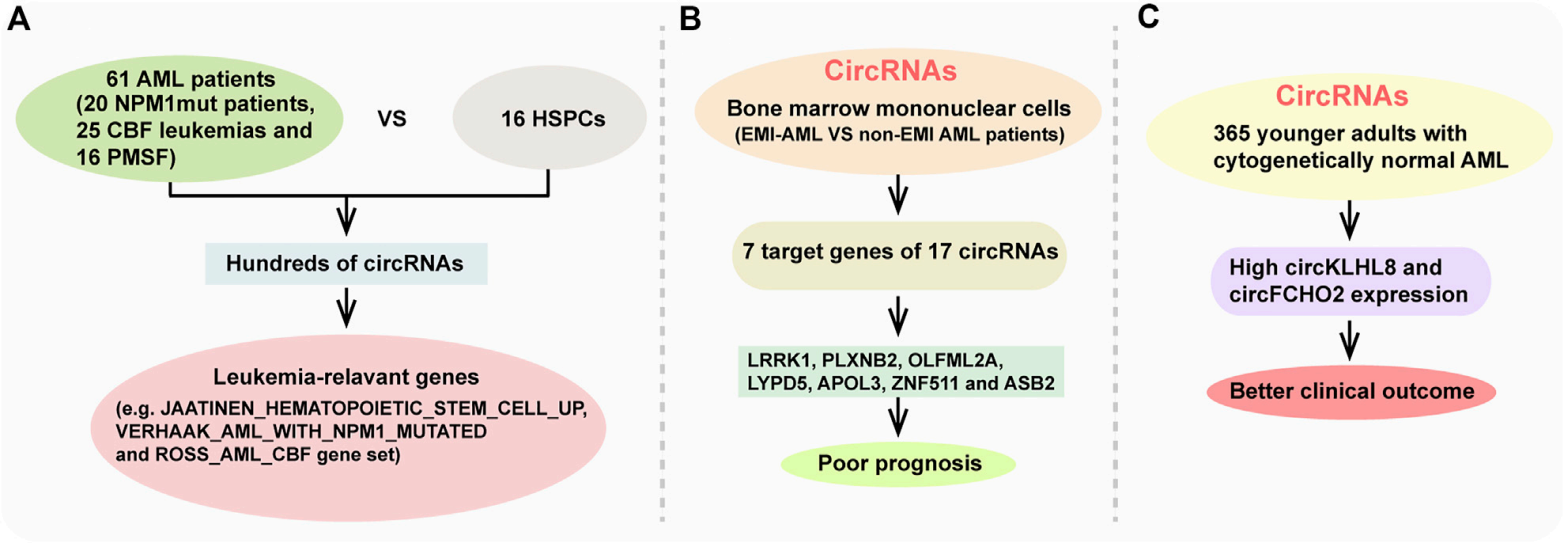
CircRNAs in AML and their association with AML phenotype. **(A)** AML-related circRNA expression patterns are enriched for leukemia-relevant gene. **(B)** seven target genes of 17 circRNAs (LRRK1, PLXNB2, OLFML2A, LYPD5, APOL3, ZNF511, and ASB2) indicated a poor prognosis. **(C)** High circKLHL8 and circFCHO2 expression were independently associated with better clinical outcome of cytogenetically normal AML patient.

CircRNAs was first reported in viroids by Sanger et al., in 1976 (Sanger et al., 1976). With the development of high-throughput sequencing and increased research interest in circRNAs, many bioinformatics tools have been improved to study circRNAs in the past few years (Wang Y. et al., 2020; Rbbani et al., 2021; Yang et al., 2021). Especially, accumulating evidence demonstrates that circRNA expression is deregulated in AML compared with healthy control and reveals AML subgroup-specific signatures (Li W. et al., 2017; Chen H. L. et al., 2018; Lv et al., 2018; Lux et al., 2021; Wang J. H. et al., 2021). For instance, Lux et al. reported that hundreds of circRNAs were differentially expressed between 61 AML patients (including 20 *NPM1mut* patients, 25 CBF leukemias and 16 patients with mutations in splicing factors (PMSF)) and 16 healthy hematopoietic stem and progenitor cell samples (HSPCs) through using ribosomla RNA-depleted RNA sequencing (Lux et al., 2021) (Figure 3A). Their results showed that circRNA expression patterns are distinct in AML subgroups compared with healthy HSPCs. Many circRNA isoforms were deregulated in only one of the AML subgroups with 40%, 51% and 24% of the differentially expressed circRNAs in *NPM1mut*, CBF leukemia and PMSF, repecstively. Their results also showed that AML-related circRNA expression patterns are enriched for leukemia-relevant genes, such as JAATINEN_HEMATOPOIETIC_STEM_CELL_UP gene set, VERHAAK_AML_WITH_NPM1_MUTATED gene set and ROSS_AML_CBF gene set (Figure 3A).

AML can develop as myobasts infiltrate into organs and tissues anywhere other than the bone marrow, which called extramedullary infiltration (EMI), revealing a poor prognosis. Through comparing differentially expressed circRNAs in bone marrow mononuclear cells between EMI-AML and non-EMI AML patients, they found that seven target genes of 17 circRNAs (LRRK1, PLXNB2, OLFML2A, LYPD5, APOL3, ZNF511, and ASB2) revealed a poor prognosis (Lv et al., 2018) (Figure 3B). Through analyzing whole-transcriptome profiling of 365 younger adults with cytogenetically normal AML, another study identified three different circRNA expression-based clusters with distinct clinical and molecular characristics such as somatic mutations, differences in age and white blood cell count. They found that high circKLHL8 and circFCHO2 expression were independently associated with better clinical outcome of cytogenetically normal AML patients (Papaioannou et al., 2020) (Figure 3C). Above all, these circRNAs sequencing results highlight a potential involvement of circRNAs in the pathogenesis of AML. However, most researches have applied bone marrow samples, and only a few used peripheral blood samples. Correlative researches between bone marrow samples and peripheral blood are also limited.

## CircRNAs regulte cell differentiation, cell cycle progression, and cell proliferation

**FIGURE 4:**
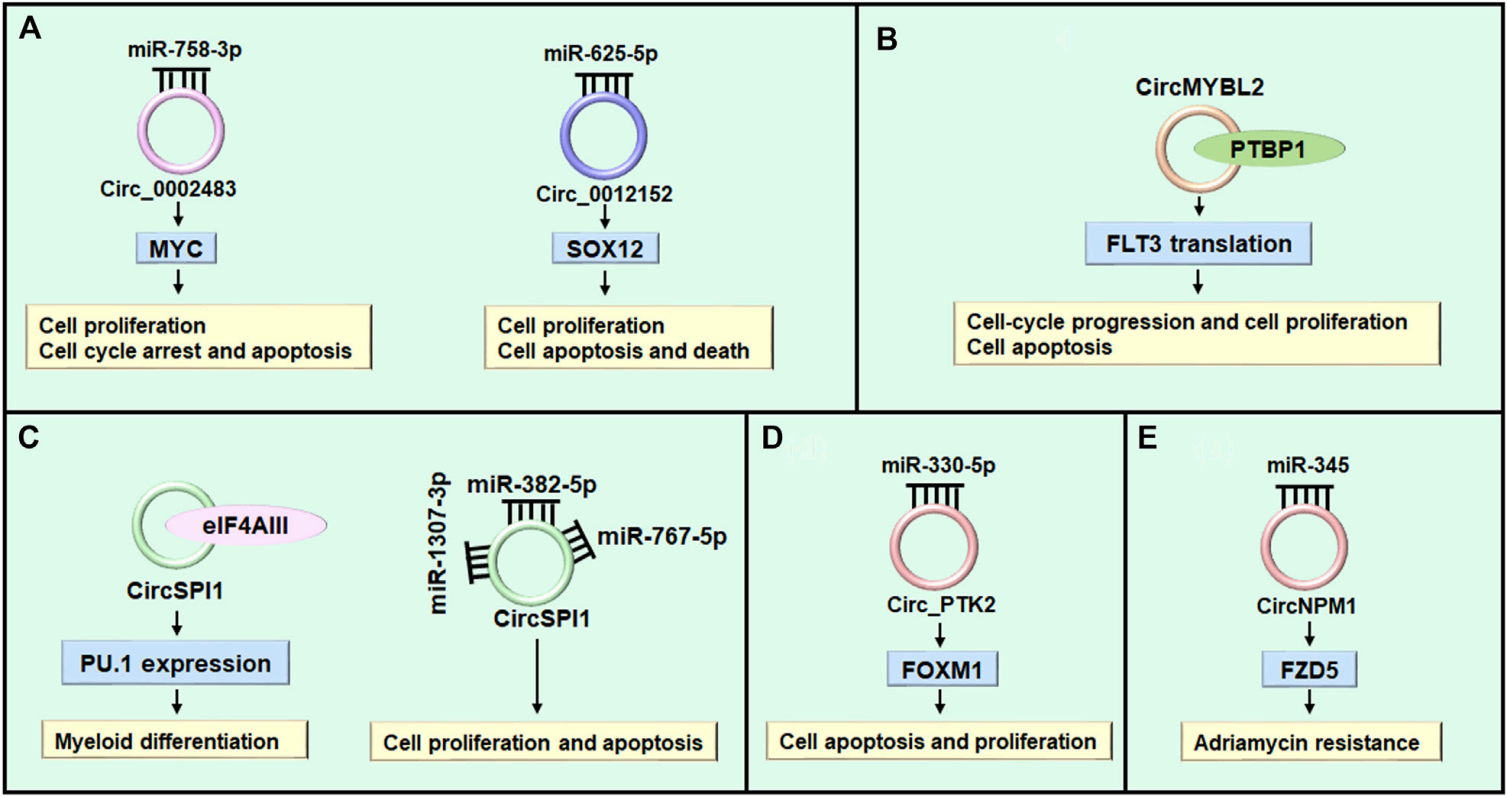
The role of circRNAs in AML. **(A)** Circ_0002483 and circ_0012152 regulate cell proliferation through sponging miRNAs. **(B)** CircMYBL2 modulates cell proliferation through regulating RBP (e.g. PTBP1). **(C)** CircSPI1 regulates myeloid differentiation of AML cells through interacting with the translation initiation factor eIF4AIII to inhibit PU.1 expression at the translation level. While, it regulates cell proliferation and apoptosis through sponging miR-1307-3p, miR-382-5p, and miR-767-5p. **(D)** CircPTK2 regulates cell apoptosis through modulating miR-330-5p/FOXM1 axis. **(E)** CircNPM1 reveals adriamycin resistance through regulating miR-345/FZD5 axis.

AML is characterized by aberrant differentiation and abnormal clonal expansion of myeloid blasts (Newell and Cook 2021; Xiang et al., 2022). It has been reported that dysregulated circRNAs can regulate cell differentiation, cell cycle progression and cell proliferation through acting as microRNA sponges in various diseases (Xiao et al., 2021; Shi et al., 2022; Wang et al., 2022). The myelocytomatosis oncogene (MYC) is a typical leukemia-associated transcription factor and plays important role in leukemic cell growth, AML cell proliferation and apoptosis (Beyer et al., 2019; Li et al., 2020c). Hsa_circ_0002483 (circ_0002483) expression was increased in AML patients and cells (Table 2). Knockdown of circ_0002483 inhibited AML cell proliferation and facilitated cell cycle arrest and apoptosis by regulating miR-758-3p/MYC axis (Xiao et al., 2021) (Figure 4A). Shang et al. found that the expression of circ_0012152 was enhanced in AML tissues and cells, circ_0012152 knockdown inhibited cell proliferation, induced cell apoptosis and facilitated death in AML cells by regulating miR-625-5p/SOX12 axis (Shang et al., 2021) (Figure 4A). In addition, Lux et al. reported that circBCL11B exclusively expressed in AML patients but not detected in healthy hematopoietic stem and progenitor cell samples, inhibition of circBCL11B suppressed leukemic cell proliferation and led to enhanced cell death of leukemic cells (Lux et al., 2021). However, the molecular mechanisms of circBCL118-mediated function in AML need to be further investigated in future studies. Except for acting as microRNA sponges, circRNAs interacting with RNA binding proteins functionally to exhibit their roles in various diseases (Sun et al., 2019; Shen et al., 2020; Zhang Y. N. et al., 2020). While, the study about circRNAs interacting with RNA binding proteins to exert their functions in AML was limited. Sun et al. reported that circMYBL2 expression to be about 5-fold higher in AML patient samples with FLT3-ITD mutations (FLT3-ITD^+^) compared with those without FLT3-ITD mutation (FLT3-ITD^-^). CircMYBL2 suppressed cell apoptosis, increased cell proliferation, and promoted cell-cycle progression in FLT3-ITD^+^ leukemic cells but not FLT3-ITD^-^ cells. Mechanistically, it increased translation of FLT3 kinase by promoting the PTBP1 binding to FLT3 messenger RNA (Sun et al., 2019) (Figure 4B). In addition, some circRNAs exert different biological functions through different mechanisms in various diseases (Xing et al., 2020; Wang X. L. et al., 2021; Yang Z. G. et al., 2022). Overexpression of circ-FOXO3 suppressed cell growth, migration and invasion through sponging miR-23 in esophageal squamous cell cancer (Xing et al., 2020), while circ-FOXO3 relieved blood-brain barrier by sequestering mTOR and E2F1 in ischemia/reperfusion injury (Yang Z. G. et al., 2022). While in AML, Wang et al. reported that silencing circSPI1 decreased myeloid differentiation of AML cells through interacting with the translation initiation factor eIF4AIII to inhibit PU.1 expression at the translation level. While, knockdown of it specially reduced cell proliferation and apoptosis through interacting with miR-1307-3p, miR-382-5p, and miR-767-5p (Wang X. L. et al., 2021) (Figure 4C).

## CircRNAs regulte cell apoptosis

Apoptosis, or programmed cell death, plays a key role in the development and homeostasis of the hematopoietic system (Testa and Riccioni 2007; Testa 2010). Although there are many factors contributing to the hematopoetic cell homeostasis, apoptotic machinery seems to have an important role (Droin et al., 2013). Recent studies indicated that circRNAs play vital role in cell apoptosis through sponging micrioRNAs in AML (Fan et al., 2018; Wu et al., 2018; Zhang L. et al., 2020; Wang X. L. et al., 2021; Xiao et al., 2021; Yi et al., 2021). Forkhead box M1 (FOXM1) functioned as an oncogene in cancers and can be regulated by multiple microRNAs in mang maliganancies (Gartel 2017; Hamurcu et al., 2021; Xing et al., 2021). While in AML, suppression of highly expressed-circ_PTK2 induced apoptosis and inhibited proliferation of AML cell by regulating miR-330-5p/FOXM1 axis (Yi et al., 2021) (Table 2) (Figure 4D). Hsa_circ-0000370 facilitated cell viability and inhibited apoptosis of FLT3-ITD-positive AML cells *via* modulating miR-1299 and S100 calcium-binding protein A7A expression (Zhang L. et al., 2020). CircRNA_100290 promoted cell proliferation and suppressed apoptosis in AML cells by regulating miR203/Rab10 axis (Fan et al., 2018). Furthermore, circRNA-DLEU2 was upregulated in AML tissues and cell, which accelerated AML cell proliferation and suppressed cell apoptosis through inhibiting miR-496 and facilitating PRKACB expression (Wu et al., 2018). In addition, Wang et al. reported that circ_0009910-containing exosomes regulated proliferation, apoptosis and cell cycle progression of AML cells partially by miR-5195-3p and GRB10 (Wang D. et al., 2021).

## Relation between circRNAs and drug resistance in acute myeloid leukemia

Drug resistance is one of the key factors that lead to the failure of AML chemotherapy (Bester et al., 2018). Various genes and non-coding RNAs are participated in the development of drug resistance in AML (Tian et al., 2017; Xu et al., 2017; Hu et al., 2018; Gebru and Wang 2020; Chen X. et al., 2021; Kirtonia et al., 2022). Non-coding RNAs, such as microRNAs and lncRNAs, are regarding as vital players in regulating drug resistance, and their targeting provides avenues for the development of new treatment choices (Tian et al., 2017; Bester et al., 2018; Wang C. et al., 2020; Kirtonia et al., 2022). Nevertheless, studies on the potential involvement of aberrant expressed circRNAs in drug resistance of AML are just appearing. Ding et al. reported that circNPM1 increased adriamycin resistance in AML through regulating the miR-345/FZD5 pathway (Ding et al., 2021) (Figure 4E). Similarly, Shang et al. found that circPAN3 was increased in refractory and recurrent AML patient tissues and doxorubicin-resistant THP-1 AML cell lines than non-transformed tissue and THP-1 AML cell lines. Mechanistically, circPAN3 could be an important mediator of chemoresistance in AML cells by regulating miR-153-5p/miR-183-5p-XIAP (X-linked inhibitor of apoptosis) axis (Shang et al., 2019). Moreover, miR-153-5p and miR-183-5p were revealed to interact with XIAP, which has been indicated as a drug resistance gene in AML (Katragadda et al., 2013). In addition, overexpression of circPVT1 has also been found to involve in resistance to vincristine in AML (L'Abbate et al., 2018), and knockdown of fusion circM9 revealed enhanced sensitivity to anti-leukemic drugs (Guarnerio et al., 2016). These results suggest that circRNAs can potentially be applied to reverse drug resistance. However, the relation between circRNAs and other drugs in AML needs to be further investigated.

In conclusion, circRNAs play important role in regulating cell differentiation, cell cycle progress, proliferation and apoptosis, as well as involve in drug resistance through acting as microRNA sponges or interacting with RNA binding proteins in AML. As discussed in above, circRNAs also can regulate gene transcription and serve as translation template to exert their function. However, whether circRNAs exert their function through regulating gene transcription and serving as translation template in AML need to be further explored.

## Clinical significance of circRNAs in acute myeloid leukemia

**FIGURE 5:**
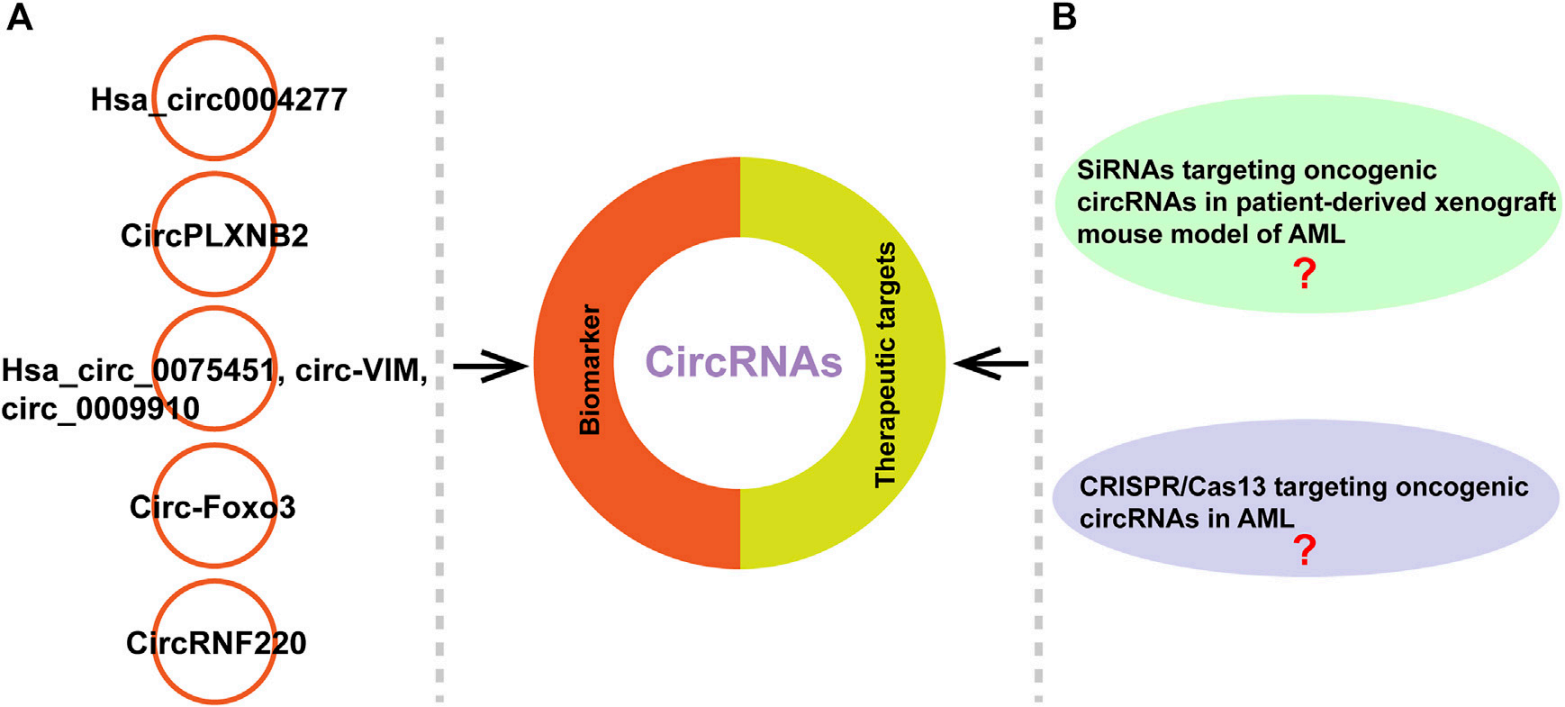
CircRNAs can serve as biomarkers or therapeutic targets. **(A)** Examples of circRNAs as potential biomarkers in AML. **(B)** The studies about siRNAs targeting oncogenic circRNAs in patient-derived xenograft mouse model of AML and CRISPR/Cas13 targeting oncogenic circRNAs in AML remain unclear.

CircRNAs have the potential to be diagostic and prognostic biomarkers, and therapeutic targets because that they are highly stable, cell- and tissue-specific expressed, and their expression levels often associated with clinical and pathological characteristics (D'Ambra et al., 2019; Li et al., 2020b; Wang Y. et al., 2020). Different molecular-based biomarkers such as cytogenetics, epigenetics, genetics, noncoding RNAs and protemocis have been well-documented in AML (Trino et al., 2018; Thakral et al., 2020; Ribeiro et al., 2021; Kirtonia et al., 2022; Wiatrowski et al., 2022). CircRNAs act as tumor suppressors or oncogenes to involve in the development of various diseases such as AML and are becoming new diagnostic and prognostic biomarkers (Zhou et al., 2020; Issah et al., 2021; Singh V. et al., 2021) (Table 3). Li et al. reported that hsa_circ0004277 might be a potential diagnostic marker through evaluating its expression in 115 AML patients samples and increasing level of hsa_circ0004277 was associated with successful chemotherapy (Li W. et al., 2017) (Figure 5A). Lin et al. found that enhanced circPLXNB2 levels were related to an obviously shorter overall survival and leukaemia-free survival of patients with AML. Their study highlights the potential of circPLXNB2 as a novel prognostic marker and therapeutic target for AML in the future (Lin et al., 2021) (Figure 5A). In other studies, they found that hsa_circ_0075451, circ-VIM and circ_0009910 can serve as important prognostic factor in AML, respectively (Ping et al., 2019; Yi et al., 2019; Wang J. H. et al., 2021). Furthermore, Zhou et al. reported that circ-Foxo3 and Foxo3 expressed low in AML patients compared to control group and patients with high expression of Foxo3 often revealed a trend of better prognosis (Zhou et al., 2019) (Figure 5A). In addition, Liu et al. recently found that circRNF220 was specifically enriched in the peripheral blood and bone marrow of pediatric patients with AML. CircRNF220 could distinguish AML from acute lymphoblastic leukemia and other hematological malignancies with high sensitivity and specificity (Figure 5A). CircRNF220 expression independently predicted prognosis, while high expression of circRNF220 was unsuitable prognostic marker for relapse (Liu X. et al., 2021) (Figure 5A).

**TABLE 3:** Clinical significance of reported circRNAs in AML.

CircRNA	Parent gene	Expression	Clinical application	References
CircRNF220	RNF200	Up	Prognostic marker	Liu X. et al. (2021)
Hsa_circ_0075451	GMDS	Up	Prognostic factor	Wang J. H. et al. (2021)
CircPLXNB2	PLXNB2	Up	Prognostic marker	Lin et al. (2021)
Circ-VIM	Vimentin	Up	Diagnostic/prognostic biomarker	Yi et al. (2019)
Circ-FOXO3	FOXO3	Down	Diagnostic marker	Zhou et al. (2019)
Hsa_circ_0009910	MFN2	Up	Prognostic biomarker and therapeutic targets	Ping et al. (2019)
Circ-ANAPC7	ANAPC7	Up	Promising biomarker	Chen H. L. et al. (2018)
Hsa_circ_0004277	WDR37	Down	Diagnostic marker and treatment target	Li W. et al. (2017)

Increasing reports in patient-derived xenograft mouse model indicated that the siRNAs specifically targeting oncogenic circRNAs can effectively suppress tumor growth (Meng et al., 2018; Zhang et al., 2019; Du et al., 2022). Meng et al. found that silencing of si-circ-10720 *via* intravenous injection inhibited the promotive effect on tumor growth and metastasis in a mouse hepatocellilar carcinoma model (Meng et al., 2018). Another study reported that knockdown of circNRIP1 using siRNA suppressed the proliferation, migration and invasion of gastric cancer (GC) cells *in vitro* and blocked tumor growth in GC-patient-derived xgenograft mouse model (Zhang et al., 2019). Recently, Du et al. reported that poly (β-amino esters)-delivered circMDK siRNA significantly inhibited the growth of hepatocellular carinoma through reducing the expression of ATG16L1 in patient-derived xenograft mouse model, suggesting that the oncogenic circMDK may be a potential treatment targtet for hepatocellular carinoma (Du et al., 2022). An interference RNA can be designed to precisely target the unique back-spliced junction of oncogenc circRNA in cancers in order to eliminate the possibility of interference with the expression of parent linear mRNA. In AML, currently, some circRNAs have been reported as oncogenic circRNAs (Ping et al., 2019; Zhang R. et al., 2020; Wang X. L. et al., 2021). Ping et al. found that circ_0009910 acting as oncogene in AML patients and knockdown of it suppressed AML cell proliferation and resulted in cell apoptosis (Ping et al., 2019). Another study reported that circRNF12 as an oncogene in blood of AML patients and interference of it reduced the migration and invasion ability of AML cells (Zhang R. et al., 2020). Wang et al. found another circRNA, circSPl1 also as an oncogene in AML, evidenced by the results that knockdown of circSPl1 induced apoptosis of AML cells (Wang X. L. et al., 2021). Although some circRNAs acting as oncogene has been reported in AML, the study about siRNAs targeting oncogenic circRNAs in patient-derived xenograft mouse model of AML needs to be explored in the future researches (Figure 5B). In addition, it has been reported that CRISPR-Cas13 system can be applied to knock down circRNAs, without any influence on related mRNAs (Koch 2021). This method has been used to few studies. For instance, Li et al. reported that knockdown circFAM120A (oncogenic circRNA) by CRISPR-RfxCas13d system promoted cell proliferation by inhibiting FAM120A from binding the translation inhibitor IGF2BP2 in 293FT cells (Li S. Q. et al., 2021). Ishola et al. found that knockdown of hsa_circ_0000190 using CRISPR/Cas13a inhibited tumor growth *in vivo* non-small cell lung cancer xenograft model (Ishola et al., 2022). However, the study about CRISPR/Cas13 targeting oncogenic circRNAs in AML needs to be investigated in the future (Figure 5B).

## Conclusion and perspective

AML is a malignant tumor characterized by the accumulation and clonal expansion of the immature myeloid hematopoietic cells in the bone marrow, with rising morbidity and mortality (Liu et al., 2019). Although advances in AML molecular characterization and targeted methods, most AML cases still lack therapeutically actionable targets and long-term survival remains low (Decroocq et al., 2022; Pabon et al., 2022). Therefore, it is necessary to discover new biomarkers for prognostication, diagnosis, and therapeutic targets of AML to explore more effective surveillance and treatment programs. It has been reported that circRNAs could regulate cell differentiation, cell cycle progress, proliferation and apoptosis, as well as involve in drug resistance in AML through acting as microRNA sponges or interacting with RNA binding proteins. However, whether circRNAs exhibit their biological function through regulating gene transcription or serving as translation template in AML need to be further investigated. Moreover, the accurate mechanism of modulation of circRNAs expression in AML is not well researched. It is not clear if abnormal circRNA expression is central event in leukemogenesis or an epiphenomenon. Most researches have applied bone marrow samples, and only a few used peripheral blood samples. Correlative researches between bone marrow samples and peripheral blood are also limited.

Recently, Qu et al. reported that circRNA vaccine successfully elicited potent neutralizing antibodies and T cell response by encoding the trimeric receptor-binding domain of SARS-CoV-2 spike protein (Qu et al., 2022). Their results suggested that the synthesis of translatable circRNAs is of great value in the field of biomedicine. Moreover, Chen et al. recently found high-efficiency method to enhance circRNA protein yields by several hundred-fold by optimizing five functional elements controlling circRNA translation including IRES, 5′ and 3’ UTRs, vector topology and synthetic aptamers (Chen R. et al., 2022). Their results enable potent and durable protein production by translatable circRNA *in vivo*. However, whether translatable circRNAs could applied to the treatment of AML required to be further investigated. Furthermore, increasing evidence indicates the siRNAs specifically targeting oncogenic circRNAs can effectively suppress tumor growth in patient-derived xenograft mouse model (Huang et al., 2020; Yang et al., 2020; Liang et al., 2021). However, siRNAs targeting oncogenic circRNAs in patient-derived xenograft mouse model of AML needs to be explored in the future study. In addition, it has been reported that CRISPR-Cas13 sysrem can be used to knock down circRNAs to explore the function of circRNAs (Li S. Q. et al., 2021; Ishola et al., 2022). However, the study about CRISPR/Cas13 targeting oncogenic circRNAs in AML needs to be investigated in the future.

Our understanding of the metabolism and transport of circRNA within and outside the cell is also lacking. It has been reported that excessive circRNAs are transported out of the cell in exosomes (Li Y. et al., 2015). This is also of great interest because it is well documented in other cancers (Wang et al., 2019; Pan et al., 2022; Yang C. et al., 2022). However, in AML, exosomal circRNAs are few been explored. The use of exosomal cirRNAs in regulating bone marrow microenvironment and extreamedullary infiltration of leukemia cells can be an interest field to research.

In summary, although great progress of circRNAs in AML has been achieved, substantial efforts are still needed to find whether circRNAs exert their biological function by other mechainsms such as regulation of gene transcription or serving as translation template in AML. It is also urgent that scientists study the machineries regulating circRNAs fate, the downstream effectors of circRNAs modulatory networks, and the clinical application of circRNAs in AML. Better understanding of these will promote our knowledge of circRNAs in AML biology and the development of circRNAs-based diagnosis, prognosis and therapeutic methods for AML.
